# Interpretable Clinical Decision Support System for Audiology Based on Predicted Common Audiological Functional Parameters (CAFPAs)

**DOI:** 10.3390/diagnostics12020463

**Published:** 2022-02-11

**Authors:** Mareike Buhl

**Affiliations:** 1 Medizinische Physik, Carl von Ossietzky Universität Oldenburg, 26111 Oldenburg, Germany; mareike.buhl@uni-oldenburg.de; 2 Cluster of Excellence Hearing4all, 26111 Oldenburg, Germany

**Keywords:** CDSS, audiology, precision medicine, interpretability, machine learning, expert knowledge

## Abstract

Common Audiological Functional Parameters (CAFPAs) were previously introduced as abstract, measurement-independent representation of audiological knowledge, and expert-estimated CAFPAs were shown to be applicable as an interpretable intermediate layer in a clinical decision support system (CDSS). Prediction models for CAFPAs were built based on expert knowledge and one audiological database to allow for data-driven estimation of CAFPAs for new, individual patients for whom no expert-estimated CAFPAs are available. Based on the combination of these components, the current study explores the feasibility of constructing a CDSS which is as interpretable as expert knowledge-based classification and as data-driven as machine learning-based classification. To test this hypothesis, the current study investigated the equivalence in performance of predicted CAFPAs compared to expert-estimated CAFPAs in an audiological classification task, analyzed the importance of different CAFPAs for high and comparable performance, and derived explanations for differences in classified categories. Results show that the combination of predicted CAFPAs and statistical classification enables to build an interpretable but data-driven CDSS. The classification provides good accuracy, with most categories being correctly classified, while some confusions can be explained by the properties of the employed database. This could be improved by including additional databases in the CDSS, which is possible within the presented framework.

## 1. Introduction

Clinical decision support systems (CDSS) provide the potential to improve objectivity in clinical decision-making, e.g., by providing clinical experts with probabilities for medical findings or diagnoses which are based on large amounts of patient data [1,2]. However, CDSS are not yet widely adopted because they lack integration into the decision-making process of clinical experts [3], act as black boxes whose functionality cannot be interpreted by the experts [1], or lack integration of different clinical data sources [4]. Therefore, CDSS need to be developed in collaboration with experts [5,6,7]. An ideal CDSS should provide interpretability to experts [1,4,8] and must exploit data from different clinical databases.

A CDSS developed in collaboration with experts can combine the advantages of expert knowledge and automatic prediction or classification [7,9]. If an expert is highly experienced, her or his knowledge is highly developed from previous patients, and many connections between different patient cases may be implicitly available. However, it takes time, work experience and effort to obtain a high degree of experience, and subjective influences are possible. In contrast, CDSS perform “objective” clinical decision making, i.e., their decision making depends on how the system was trained and which data were available for training [2]. By using large amounts of data, precise classifications become possible that rely on better statistics than a human expert could achieve with a limited number of seen patients. In addition, new relationships in the data could be discovered which the human expert did not know before [10]. A disadvantage is that the CDSS can only represent what it has learned in training, and therefore, it could miss some special cases. In addition, CDSS are not able to use subjective impressions about patients. If a system provides interpretability, e.g., in terms of explanations of how the system works, appropriate visualizations or uncertainty measures, experts can gain trust and acceptance [1,3,11,12]. Therefore, the interplay between human experts and CDSS should provide optimal benefits towards precision medicine.

To further the usage and exploitation of “Big Data” for achieving high accuracy, clinical data distributed over different local databases and with potentially different content—e.g., different parameters or measurements being collected at a respective clinic—need to be jointly analyzed and combined. This could be achieved by introducing a common data format, as suggested, for example, in [2]. Even in one medical discipline, this would involve adapting all databases to a defined format (and selection of measurements) on which every location agrees, which is difficult to realize on the basis of single projects. Coordinated initiatives such as HiGHmed [13] work towards standardized data formats and data integration centers for medical data. By separating knowledge and information, as well as technical and domain content, interoperable information systems can be obtained [13,14]. The domain knowledge is incorporated in so-called archetypes, which need to be defined for different medical fields and applications [14]. This approach was, for example, successfully implemented in infection control, where data could be jointly analyzed in a multicentre study after transforming it to archetypes [15].

In the field of audiology, the aim is to characterize patients’ hearing impairment (e.g., sensorineural, conductive, or mixed hearing loss) and to suggest an appropriate treatment (e.g., provision with a hearing aid (HA) or implantation of a cochlear implant (CI)) to compensate for the hearing loss [16,17]. For this purpose, audiological measurements are conducted; these measurements differ across clinics but also depend on the target group of the respective institution. For example, audibility is assessed by a pure-tone audiogram in most databases, but communication abilities are assessed by the use of different speech tests based on words or sentences. According to German clinical guidelines [18], hearing device indication criteria are based on the Freiburg monosyllabic speech test [19]. For the assessment of speech understanding in noise or hearing aid benefit, sentence tests in noise such as the matrix sentence test [20,21,22,23] or the Goettingen sentence test (GÖSA; [24]) are used.

With respect to machine learning methods for characterizing hearing impairment or suggesting a treatment in the direction of a CDSS, few approaches exist for audiology. For example, Sanchez-Lopez et al. [25,26] investigated the classification of hearing-impaired patients into auditory profiles describing distortions related to audibility or not based on previously-published research data sets. In addition, different approaches of CDSS exist that consider single aspects of audiology, such as, for example, a CDSS for tinnitus diagnosis and therapy [27], for idiopathic sudden hearing loss [28], or for the selection of in-the-ear (ITE) vs. behind-the-ear (BTE) hearing aids [29]. Moreover, [27] considered the aspect of interpretability and both [27,28] worked with data from (country-specific) electronic health records. To the best of our knowledge, no CDSS is available in the field of audiology that attempts to cover a broad range of audiological findings and treatment recommendations (diagnostic cases) as well as of test batteries of audiological measures conducted in different clinics.

**Figure 1:**
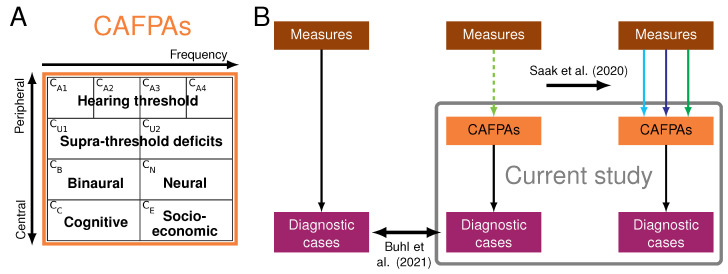
(**A**) Definition of CAFPAs. From left to right, increasing frequency is represented, and from top to bottom, peripheral to central aspects are represented. (**B**) Relationships between previous work and current study. Black arrows represent a classification, light green (dashed) arrows represent expert knowledge, and light blue, blue, and green arrows represent different CAFPA prediction models (lasso regression, elastic net, and random forest, respectively). Buhl et al. [32] compared classification into diagnostic cases based on measures vs. CAFPAs (left and middle part). Saak et al. [33] derived regression models for CAFPA prediction based on the expert link between measures and CAFPAs (middle and right part). The current study (grey box) investigates the application of predicted CAFPAs in the CDSS as compared to classification based on expert-estimated CAFPAs.

To facilitate a CDSS for audiology representing the audiological decision-making process, including interpretability of the system and integration of different audiological databases, Buhl et al. [30] introduced the Common Audiological Functional Parameters (CAFPAs) as abstract and interpretable representations of audiological knowledge. The CAFPAs were defined in discussions with experts; in the CAFPAs, ten functional aspects describe the human auditory system independent from the exact choice of audiological measures. The CAFPAs are designed to cover all relevant aspects which are important to characterize hearing loss or suggest a treatment recommendation. Figure 1A gives an overview of the definition of CAFPAs. The CAFPAs were designed to be used as an intermediate layer between measures and diagnostic cases in the CDSS.

For the purpose of linking the CAFPAs to audiological data (measurements and diagnostic cases), Buhl et al. [31] conducted an expert survey. Thereby, CAFPAs and diagnostic cases were estimated for given patient cases from a pre-clinical database from Hörzentrum Oldenburg. Based on this data set, Buhl et al. [32] showed that a similar classification performance is obtained using expert-estimated CAFPAs, compared to directly using the audiological measurements (cf. Figure 1B, left and middle part). However, the classification of [32] has, thus far, not been applicable to patients other than those contained in the expert-labeled data set, because no quantitative link between measurement outcomes and CAFPAs has been available. Hence, CAFPAs could not be estimated for new patients. Therefore, as a next step towards a CDSS operable for individual patients, Saak et al. [33] established regression models that allow automatic prediction of CAFPAs given the measurement outcomes (cf. Figure 1B, middle and right part). Three different models (lasso regression, elastic net, and random forest) were investigated in [33], and it was shown that all models provide adequate to good predictive performance. In addition, measurements employed in the prediction of CAFPAs were analyzed by means of feature importance, which provided interpretability of the CAFPA prediction and revealed audiologically plausible relationships between measurement outcomes and CAFPAs [33].

Therefore, by combining these previous steps [32,33], the current study aims at exploring whether a CDSS can be constructed for audiology which is as interpretable as expert knowledge-based classification and as data-driven as machine learning-based classification. In the CDSS, CAFPAs are to be estimated from the available audiological measurements, and classification of diagnostic cases (audiological findings or treatment recommendations) needs to be performed based on predicted CAFPAs (cf. Figure 1B, right part). In addition, the interpretability of the CDSS needs to be assured, which can partly be combined from the interpretability aspects from the classification of [32] and CAFPA prediction of [33].

The research hypothesis of the current paper is that the combination of CAFPAs and statistical classification enables an interpretable but data-driven CDSS to be built. To test this hypothesis, the following steps were taken:

1. Model-predicted and expert-estimated CAFPAs were investigated to determine whether they could provide equivalent classification performance;
2. The classification approach and evaluation was extended to applicability for individual patients, and;
3. The interpretability of the obtained CDSS was investigated.

## 2. Materials and Methods

### 2.1. Common Audiological Functional Parameters (CAFPAs)

The Common Audiological Functional Parameters (CAFPAs) were introduced by Buhl et al. [30] as an abstract and common data format for describing the hearing status of patients. The ten CAFPAs describe different functional aspects of the human auditory system (cf. Figure 1A), which were defined from literature and by discussion with experts. The CAFPAs $C_{A1}$ to $C_{A4}$ describe audibility for different frequency ranges. $C_{U1}$ and $C_{U2}$ describe supra-threshold deficits for low and high frequencies; that is, they relate to speech intelligibility and loudness perception. Binaural, neural, and cognitive properties of the auditory system are represented by $C_{B}$, $C_{N}$, and $C_{C}$, and the socio-economic status of patients is described by $C_{E}$. From left to right in Figure 1A, increasing frequencies are represented by CAFPAs, while from top to bottom, peripheral to central aspects of hearing loss are represented. CAFPAs are defined as continuous variables in the interval [0 1] with 0 representing “normal” and 1 representing “maximally impaired”. CAFPAs are graphically represented using a traffic-light color scheme from green to red. CAFPAs are to be estimated from audiological measures available in a respective clinical database while being independent of the exact choice of tests and thus providing the potential to integrate different databases [30]. Therefore, the CAFPAs are suitable as interpretable, intermediate representation between audiological measurements and diagnostic cases in a clinical decision support system for audiology [31,32].

### 2.2. Data Set

The analysis is based on a data set from Hörzentrum Oldenburg GmbH, which was described by [34]. The data set contains the following audiological measures for 595 patients with mild to moderate hearing loss: pure-tone audiogram, Goettingen sentence test (GÖSA; [24]), adaptive categorical loudness scaling (ACALOS; [35]), DemTect [36] for characterizing cognitive abilities, a verbal intelligence test (“Wortschatztest”; [37]), the Scheuch–Winkler Index (SWI; [38]) for describing socio-economic status, and subjective information about hearing problems in quiet and in noise as well as tinnitus, gender, and age. Hence, this data set contains appropriate information to estimate CAFPAs from these measures. In an expert survey, Buhl et al. [31] collected expert labels for 240 of the 595 patients (in total, 287 cases were labeled, because for an analysis of agreement, some patients were shown to several experts [31]). Given the measures for individual patients, the experts’ task was to estimate CAFPAs and to tick audiological findings and treatment recommendations from a given list. For details, please refer to [31]. The labels for diagnostic cases are assumed as the ground truth for classification in the following. For patients where multiple expert CAFPAs are available from the expert survey, CAFPAs from one expert were randomly chosen.

### 2.3. Prediction of CAFPAs

For the purpose of providing a quantitative link between audiological measures and CAFPAs, Saak et al. [33] established regression models based on the data set [34] and the expert-estimated CAFPAs from [31]. To incorporate different degrees of interpretability vs. flexibility of the models, the regression was performed using lasso regression, elastic nets and random forests [39]. Separate models were estimated for each CAFPA. Lasso regression and elastic net perform feature selection as defined by a penalty term; that is, not all audiological measures are used to predict CAFPAs. For lasso regression, some features are shrunken to zero, while for elastic net, irrelevant features are shrunken towards zero and correlated features are grouped together [39]. For random forest, multiple decision trees are combined which each consider a limited number of features. Within a decision tree, recursive binary splitting of the feature space (the audiological measures) is performed according to the respective largest error reduction. The prediction is defined as the mean of remaining features in the resulting region of the feature space. For details on the models, please refer to [33].

Overall, a good predictive performance was obtained, with very similar performance observed across different models and larger performance variation observed across different CAFPAs. The audibility-related CAFPAs $C_{A1}$ to $C_{A4}$ were the best predicted, while the worst predictive performance was obtained for the supra-threshold CAFPAs $C_{U1}$ and $C_{U2}$. Analysis of feature importance revealed which audiological measurements were used by the models for prediction of CAFPAs, and thereby contributed to the interpretability of the predictions. Some differences across models occured, but the features commonly used by all models were plausible from an audiological interpretation point of view [33].

In the following, classification will be performed on CAFPAs predicted by all three models. To generate predictions for all 240 patients that were labeled in the expert survey of [31], a 5-fold cross-validation was performed. The model-building according to the procedures from [33] was conducted five times on the respective 80% of the data, and then the remaining 20% of the patients were predicted.

### 2.4. Classification

#### 2.4.1. Expert-Estimated vs. Model-Predicted CAFPAs (Comparison Sets)

**Figure 2:**
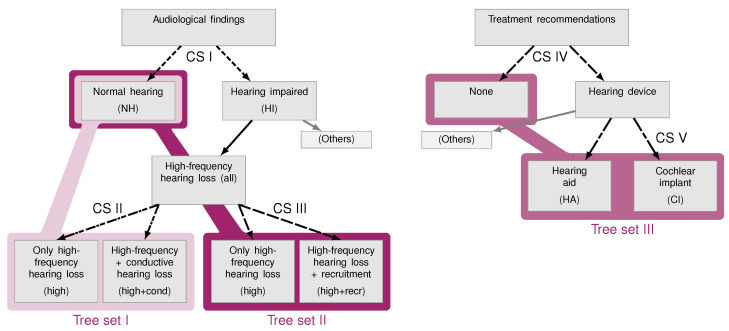
Schematic representation of comparison sets (CS) and tree sets. Different arrow line styles belong to different comparison sets of two respective categories, as described in [31,32]. Different colors show the comparison of categories performed in the respective tree set, which were derived by combination of comparison sets. Abbreviations as introduced in parentheses are used throughout the paper. This figure was adapted from Figure 2 of [31].

**Figure 3:**
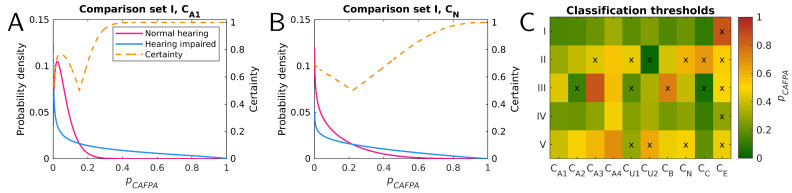
(**A**) Training distributions for $C_{A1}$ in CS I (normal hearing (magenta) vs. hearing impaired (blue)), and corresponding certainty value for different input $p_{CAFPA}$ values (orange, dashed). (**B**) As (**A**), for $C_{N}$. (**C**) Classification threshold $p_{CAFPA}$ values for all five comparison sets (rows). Thresholds correspond to intersections of training distributions, as well as to the minimum of certainty as shown in (**A**,**B**). Colors represent different $p_{CAFPA}$ values. Values lower than threshold are classified into the first category, and higher values are classified into the second category. Fields marked with “x” provide an expected value of certainty of ≤0.65 (averaged over the complete $p_{CAFPA}$ range).

To compare performance between expert-estimated and model-predicted CAFPAs, first, the classification is performed using a Bayes classifier [40] as in [32]. There, classification was performed in five binary comparison sets (CS) of two respective categories which were derived from the lists of diagnostic cases in the expert survey of [31]. These comparison sets are depicted in grey in Figure 2. For each category of each comparison set, training distributions were estimated in [31]. For the expert CAFPAs corresponding to the respective category, beta distributions were calculated (using a leave-one-out cross-validation) for each CAFPA. Figure 3A/B shows examples of training distributions for normal hearing vs. hearing impaired (CS I) for $C_{A1}$ (audibility for low frequencies) and $C_{N}$ (neural CAFPA).

In this study, the classification of expert-estimated as well as predicted CAFPAs is based on these expert training distributions, because in the use case of a CDSS, new patients would be classified using a previously trained system. For each CAFPA, the classified category is estimated by calculating the maximum of two training distributions for a given $p_{CAFPA}$ value (x-position in Figure 3A,B). Figure 3C shows resulting classification thresholds (Bayes decision boundary, [40]) for all CAFPAs and comparison sets, i.e., lower $p_{CAFPA}$ are classified to the first and higher $p_{CAFPA}$ are classified to the second category of the respective comparison set. Especially in comparison sets II and III, some CAFPAs show relatively low average certainty; that is, the training distributions for the two compared categories are similar. For classification based on all CAFPAs, the maximum is calculated based on a weighted sum of probability density values for different CAFPAs. All binary combinations of CAFPAs are used as weights for classification; that is, $2^{10}-1=1023$ combinations of the different CAFPAs being included or not are investigated. For details, please refer to [32].

The classification performance is evaluated using the Youden index *Y* as in [32]. The Youden index is calculated from sensitivity and specificity according to Equation (1), and is defined in the interval [0 1]. Sensitivity and specificity describe the proportion of correctly classified patients with respect to the first or second category of each comparison set. For all CAFPAs (expert-estimated and predicted), the expert labels are assumed as true diagnostic cases. Note that for the calculation of *Y*, only those patients that were uniquely associated by experts to the first or second category (and not to both) were taken into account.

(1) Y=Sens+Spec−1

For the comparison of expert-estimated and predicted CAFPAs, a criterion is defined that allows the investigation of CAFPA combinations (weights) that lead to high and similar performance between expert-estimated and predicted CAFPAs at the same time. This is assumed for weight vectors fulfilling Y≥0.90·max(Y), that is, those CAFPA combinations that obtain a classification performance of at least 90% compared to the maximum performance of expert-estimated or predicted CAFPAs in the respective comparison set (denoted as Y90 combinations in the following). The common high performance is then analyzed based on overlapping combinations between the expert-estimated and predicted CAFPAs of the respective model. From the relative frequency of CAFPAs in the combinations fulfilling the criterion, additional weights are derived by normalization, which are additionally used for classification (denoted as *rel-model*). Similarly, weights leading to high and common performance across CAFPAs estimated by experts and predicted by all models are investigated and additionally used for classification (denoted as *rel-all*). In summary, classification is performed in the dimensions of 5 comparison sets, 3 + 1 models/expert, 1023 + 2 weights, and 240 patients.

#### 2.4.2. Individual Patients (Tree Sets)

To achieve a CDSS applicable for individual patients, the classification was adapted in three aspects. First, the comparison sets were combined such that a classification would, for example, not stop with “hearing impaired”, but continues with finer-grained classification of the type of hearing impairment (and correspondingly for treatment recommendations). Three “tree sets” were defined this way, which are depicted in different colors in Figure 2. Second, weights of CAFPAs needed to be chosen, which happened based on the analysis performed in comparison sets (cf. Section 3.1). Third, a certainty measure was introduced for the purpose of allowing a potential user of a CDSS (expert) to interpret the classification in a statistical sense and to decide how far she or he would trust the system.

For the classification in tree sets, the classification within each comparison set was the same as described before, but in the “second layer” (comparison sets II, III, and V), only those patients were used that had been classified to the appropriate previous category before; that is, the binary decision was propagated through the tree. Thereby, within each tree set, a decision between three categories was performed (cf. Figure 2).

To investigate whether the weights derived for high and comparable classification between expert-estimated and predicted CAFPAs (cf. Section 2.4.1) were appropriate, confusion matrices and accuracy were calculated. For confusion matrices, the numbers of patients that were classified into the different categories by expert or respective model CAFPAs were estimated. Accuracy was derived from that according to Equation (2), indicating the proportion of correct classifications of all classifications, with *N* denoting the number of patients per group and *c* the index of the classified category. For graphical representation, the confusion matrices were normalized with respect to expert categories, such that the proportion of correctly classified patients can be intuitively compared across columns (cf. Section 3.2 ).

(2) $$Acc=\frac{\sum_{c}^{3}N\left(c_{class,expert}=c_{class,predicted}\right)}{\sum_{c}^{3}\sum_{c^{\prime }}^{3}N\left(c_{class,expert},{c^{\prime }}_{class,predicted}\right)}$$

The certainty measure within each comparison set is defined according to Equation (3), i.e., it describes the probability of the respective classified category *c* in relation to the sum of training distributions for given $p_{CAFPA}$ (Bayes error rate, [40]). The certainty (1-error) depends both on the training distributions (i.e., general ability to discriminate the two categories using one CAFPA) and the individual CAFPA value $p_{CAFPA}$ (i.e., relative to classification threshold as given by training distributions). The former describes the expected value of maximum certainty that can be obtained with the respective training distributions. It can be estimated by averaging the certainty across the $p_{CAFPA}$ axis [40]. Examples for $C_{A1}$ and $C_{N}$ in comparison set I are shown in Figure 3A,B. For the combination of CAFPAs, the certainty is weighted in analogy to the probabilities used for classification (all binary combinations of CAFPAs). The certainty for tree sets is estimated by propagating it through comparison sets as defined for the classification. Therefore, the certainties for the three categories of each tree set sum to 1 by definition. Due to the definition of tree sets, the certainties can be interpreted as the probability of the classified category being correct, but with a chance level of 0.5 for the respective first and 0.25 for the second and third category (e.g., for normal hearing, the “classification path” is already terminated, while for hearing impaired, a second comparison set follows).

(3) $$Cert_{c}\left(p_{CAFPA}\right)=\frac{p_{c}\left(p_{CAFPA}\right)}{\sum_{c^{\prime }}^{2}p_{c^{\prime }}\left(p_{CAFPA}\right)}$$

## 3. Results

### 3.1. Expert-Estimated vs. Model-Predicted CAFPAs (Comparison Sets)

In the first part of the analysis, classification was performed in comparison sets to investigate if good and comparable classification performance can be obtained by employing the model-predicted CAFPAs from [33] as compared to expert-estimated CAFPAs from [31], and to derive weights of CAFPAs to use in Section 3.2.

**Figure 4:**
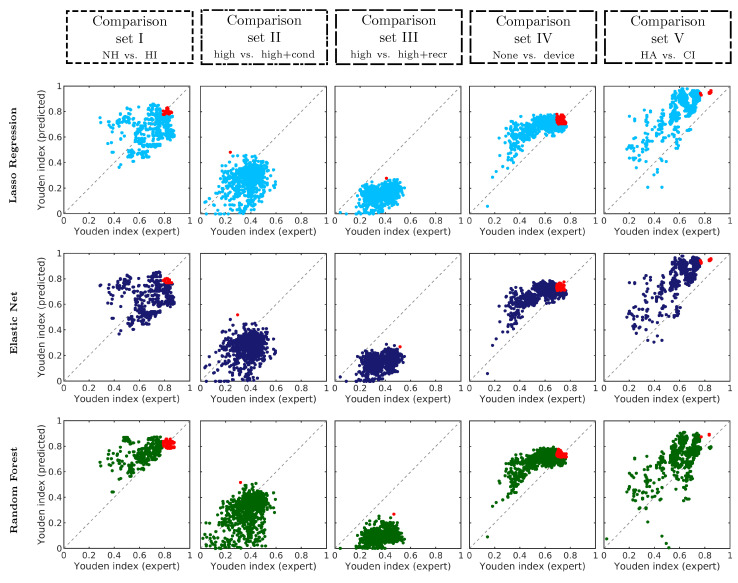
Youden index scatter plots for all 1023 binary combinations of CAFPAs. Rows and colors depict results for different CAFPA prediction models, while columns depict comparison sets. In each panel, the Youden index *Y* for predicted CAFPAs is plotted against the Youden index *Y* for expert-estimated CAFPAs. Red data points represent Y90 combinations.

Figure 4 shows the classification performance in terms of the Youden index *Y* for all 1023 binary combinations of CAFPAs. Rows depict different prediction models compared to expert-estimated CAFPAs, and columns depict different comparison sets. In the scatter plots, the diagonal represents comparable performance between expert-estimated and predicted CAFPAs, and the best performance is located in the top-right corner. Across models, the general distribution of performance is similar, while larger differences across comparison sets occur, especially in terms of the maximal possible performance, which is in line with [32].

Some bias between expert-estimated and predicted CAFPAs (for all models) occurs in comparison sets III and V. In comparison set III, predicted CAFPAs achieve lower performance than expert-estimated CAFPAs, while in comparison set V, predicted CAFPAs achieve higher performance. For all models and comparison sets, large performance variations across CAFPA combinations (defined by weights) occur regarding best performance but also comparability between expert-estimated and predicted CAFPAs. Therefore, an appropriate choice of CAFPAs is important for the application of CAFPA predictions in a clinical decision support system.

**Figure 5:**
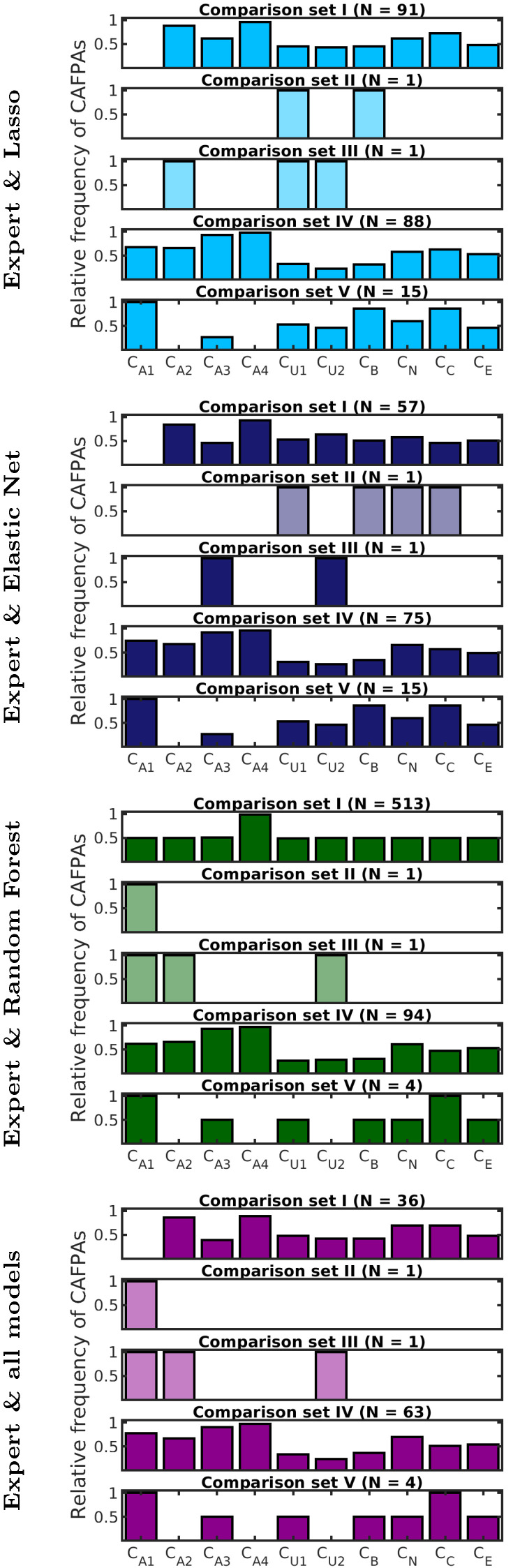
Relative frequency of CAFPAs included in Y90 combinations, common for expert-estimated CAFPAs and respective models (first three panels, colors for different models). The last panel (purple) shows the relative frequency of CAFPAs common to experts and all models. If no common weights were found, the best CAFPA combination of the respective model is depicted (lighter colors).

For the purpose of investigating the importance of different CAFPAs for different models and comparison sets, the contribution of different CAFPAs to high classification performance (Y90 combinations, red data points in Figure 4) was analyzed. Figure 5 shows the relative frequency of CAFPAs in combinations that are common between the expert-estimated CAFPAs and the respective model-predicted CAFPAs, as well as common between experts and all models. For all models, all CAFPAs contribute to high performance in at least one comparison set, and the combinations within comparison sets are similar across models.

Differences between models are visible in the number of included CAFPA combinations. For comparison set I, a medium number of Y90 combinations was found for lasso regression and elastic net, while about half of all combinations show a high performance for random forest. In all of these combinations, $C_{A4}$ is included, and all remaining CAFPAs are included in half of the Y90 combinations. This means that $C_{A4}$, describing audibility for high frequencies, could also be used alone for classification when CAFPA prediction is done with random forest (in comparison set I) and all other CAFPAs occur in varying combinations; hence, the exact choice among them is not important. In contrast, lasso regression and elastic net seem to represent distinctive information from different CAFPAs, as their relative importance differs across CAFPAs. For these models, the choice of CAFPAs is more crucial but more interpretable. However, all models agree that $C_{A4}$ (and $C_{A2}$) are most important for this comparison set, which is plausible because an audiogram at high frequencies is well able to discriminate between normal hearing and hearing-impaired cases, especially for high-frequency hearing loss, which is frequent in the considered data set.

In comparison set IV (none vs. hearing device), a noticeable high number of Y90 combinations is present for all models. Here, again, different CAFPAs contribute to high performance, but their relative importance differs, and the agreement across models is very high.

In comparison sets II and III, very few combinations are best-performing and common with experts for all models. Compared to Figure 4, this can be explained with the general lower performance of model-predicted CAFPAs as compared to expert-estimated CAFPAs. In these comparison sets, no common weights between all models were found. In this case, the best CAFPA combination of the respective model was estimated.

For the purpose of representing the importance of different CAFPAs in the classification of individual patients (cf. Section 3.2), the relative frequency of CAFPAs common between models and expert was normalized and then used as additional weights (*rel-model*) in the classification with the respective model (in addition to all binary combinations as shown before). In comparison sets II and III, the weights of best performance were used if no common combination between expert-estimated and predicted CAFPAs was available. Furthermore, the weights derived from common CAFPA combinations across all models were also used in the classification (purple in Figure 5, *rel-all*).

**Figure 6:**
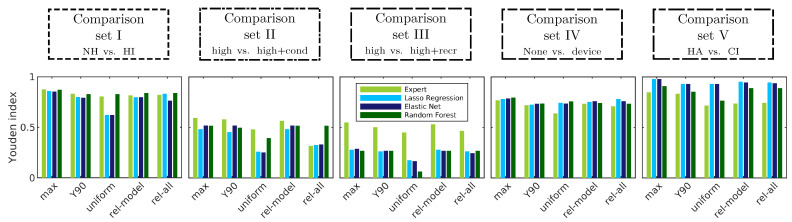
Youden index *Y* depicted for different comparison sets (panels), groups of CAFPA combinations (*x*-axis), as well as expert-estimated and model-predicted CAFPAs (colors, as introduced in Figure 4). Conditions on the *x*-axis comprise the maximum performance in the respective comparison set and model, median performance in Y90 combinations (red data points in Figure 4), and performance using *uniform*, *rel-model*, and *rel-all* weights. For classification based on expert-estimated CAFPAs, *rel-model* and *rel-all* weights were estimated based on Y90 combinations for expert classification alone; hence, these are not common weights between expert and model, as depicted in Figure 5, but are depicted in addition.

To provide an overview of classification performance in all dimensions (comparison sets, CAFPA prediction models, different combinations of CAFPAs as given by weights) and to validate classification performance using *rel-model* and *rel-all* weights derived for different models, Figure 6 summarizes the Youden index obtained for different (groups of) weights.

For all comparison sets, the maximum performance as already described is depicted to provide comparability to the other conditions. The median of Y90 performance is slightly lower than the maximum performance, which is plausible due to the design of the Y90 criterion including CAFPA combinations that lead to more than 90% of the maximum performance. The performance of different weights is depicted to investigate their applicability in the classification. *Uniform* weights—which are considered to be the baseline—achieve the lowest performance of all conditions. Comparing across weights, the model-specific *rel-model* weights achieve the highest performance in all comparison sets, as well as comparable performance to the Y90 condition. In comparison sets IV and V, the performance of *rel-all* weights is similarly high, but here, the *rel-model* weights of the different models are very similar, as seen in Figure 5. However, the expert performance in these cases differs across weights and is highest for *rel-model* weights. Furthermore, the usage of generalized weights across models (*rel-all*) depends on the robustness of their estimation, i.e., if and how many common CAFPA combinations across weights are available. Therefore, *rel-all* weights are not very robust in comparison sets II and III.

Differences across models are comparison set- and weight-dependent, as, for example, random forest performing similarly to expert-estimated CAFPAs and better than lasso regression and elastic net in comparison sets I and II; however, in comparison set IV, all models performed similarly. In comparison set V, the models even achieved higher performance than expert-estimated CAFPAs.

In summary, predicted CAFPAs by all regression models achieve a comparable performance to expert-estimated CAFPAs. However, a comparable and high performance for expert-estimated and predicted CAFPAs depends on the choice of weights defining the employed CAFPAs. In the following, the model-specific weights derived from relative frequency of CAFPAs in Y90 combinations (*rel-model*) will be considered as the most promising and robust candidate for the classification of individual patients and will be compared to a baseline of using *uniform* weights, as well as to common weights for all models (*rel-all*).

### 3.2. Individual Patients (Tree Sets)

**Figure 7:**
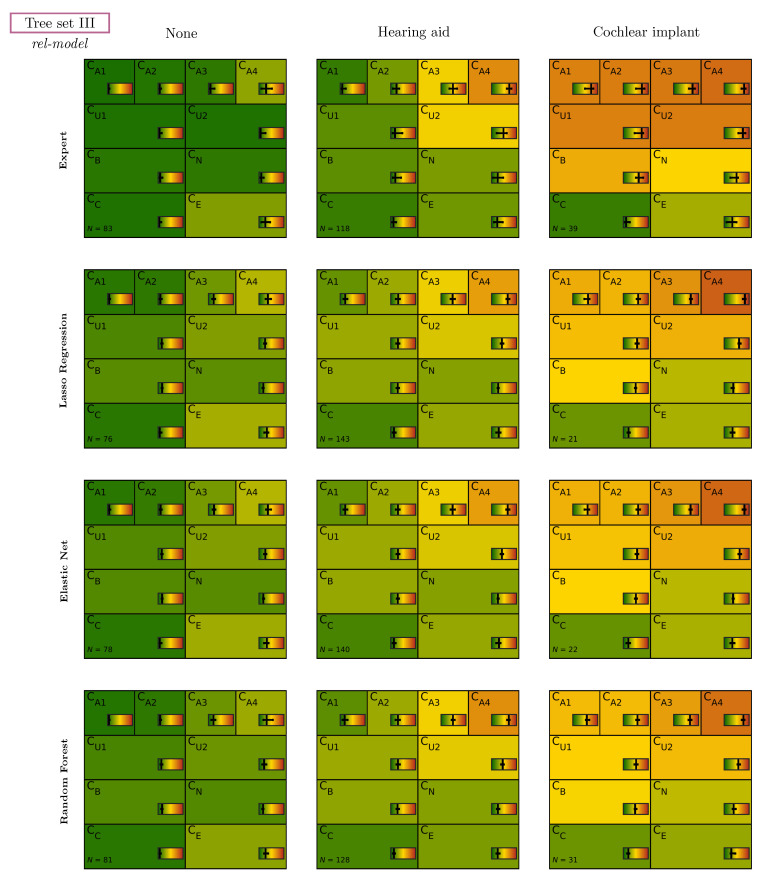
CAFPA patterns (median and interquartile ranges) of patients classified in tree set III for expert and all models (rows) using *rel-model* weights. *N* indicates the number of included patients. CAFPAs for tree sets I and II and different weights are provided in the Supplemental Materials;.

For the evaluation of individual patients, the comparison sets were combined to three tree sets, and classification was propagated through trees. Figure 7 shows median $p_{CAFPA}$ values for patients classified into the different categories of tree set III (none vs. hearing aid vs. cochlear implant) based on expert-estimated CAFPAs as well as on CAFPAs predicted by all models, and using *rel-model* weights. For all categories, plausible CAFPA patterns were obtained, with increasing $p_{CAFPA}$ values from the first to third category and with more central CAFPAs being more and more affected. Between CAFPA prediction models, the patterns are highly similar, while partly showing lower median $p_{CAFPA}$ as compared to expert-estimated CAFPAs in the cochlear implant category.

In addition, the number of patients classified into the categories differ between expert-estimated CAFPAs and different models; that is, for some patients the classification was different. The median CAFPAs for different categories of the other tree sets and weights are provided in the Supplemental Materials. Within all tree sets, distinguishable and plausible patterns were found, and different weights lead to some small differences in the numbers assigned to each category.

**Figure 8:**
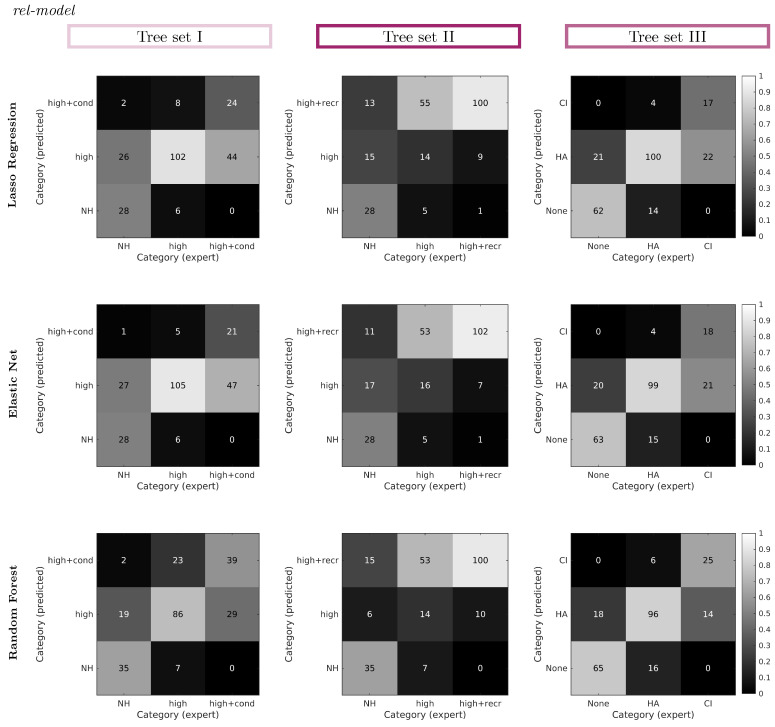
Confusion matrices of classified categories based on expert-estimated CAFPAs vs. predicted CAFPAs by the different prediction models. Numbers of patients (per expert category) are normalized within each column (represented by the grey scale), while absolute numbers are depicted as text. Different columns of the overall plot represent different tree sets, while different rows represent different CAFPA prediction models. Results are depicted for *rel-model* weights. Results for the remaining weights are provided in Table A1.

To further investigate the differences between the expert-estimated and predicted CAFPAs used for classification, Figure 8 displays confusion matrices for all models and tree sets using *rel-model* weights. In each plot, the absolute numbers of patients as classified by expert-estimated or predicted CAFPAs are represented, while the color is normalized in columns; that is, they represent the relative amount of patients classified into categories as given by expert-estimated CAFPAs. By comparing across tree sets, it can be seen that different confusions occur most often. For tree set I, patients classified to all categories using expert-estimated CAFPAs were most often classified as high-frequency hearing loss using predicted CAFPAs. For tree set II, most high-frequency hearing loss patients (expert-estimated CAFPAs) were classified as high-frequency hearing loss + recruitment by the models. For tree set III, the most prominent confusion happened between the classifications of cochlear implant (expert) and hearing aid (predicted).

Between models, confusions by lasso regression and elastic net are very similar, while random forest shows slightly reduced numbers of the most prominent confusions in each tree set as described before.

This is also reflected in the accuracies listed in Table 1, which are very similar across models (for *rel-model* weights) in tree sets I and II, while being highest for random forest in tree sets I and III. Compared across weights, accuracy is in general higher for *rel-model* and *rel-all* weights (compared to *uniform*), but in tree sets I and II, the common weights across models (*rel-all*) were estimated by very few CAFPA combinations that are not even common for all models (as described above and depicted in Figure 5).

**Figure 9:**
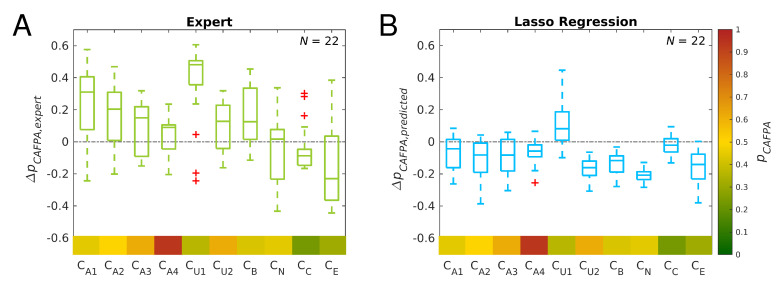
Differences $\Delta p_{CAFPA}$ between CAFPAs employed for classification and classification thresholds for all patients classified as CI by predicted CAFPAs (lasso regression) and classified as HA by expert-estimated CAFPAs in tree set III using *rel-model* weights (N=22 as depicted in Figure 8, top-right panel). Median and interquartile range are depicted. Positive values indicate classification as CI and negative values indicate classification as HA. Classification thresholds (for comparison set V, from Figure 3C) are displayed in the bottom row in the typical CAFPA color-coding. (**A**) Classification based on expert-estimated CAFPAs. (**B**) Classification based on predicted CAFPAs (lasso regression).

For a more detailed explanation for the observed confusions, Figure 9A,B shows the expert-estimated and predicted CAFPAs (lasso regression) which lead to confusion between cochlear implant and hearing aid in tree set III. By calculating the difference in classification thresholds $\Delta p_{CAFPA}$, the CAFPAs can be considered relative to the classification threshold. For expert-estimated CAFPAs, more $p_{CAFPA}$ are above the classification threshold (positive differences) and therefore classified as CI, while predicted $p_{CAFPA}$ values are mainly below the classification threshold. This explains the confusion of these categories, and shows at the same time a limitation of the current CAFPA prediction; hence, less extreme CAFPAs are predicted by lasso regression in this case. Similarly, all other confusions can be explained, as the classification is based on maximum probabilities of two compared categories, and therefore, the classification threshold determines the classified category.

**Figure 10:**
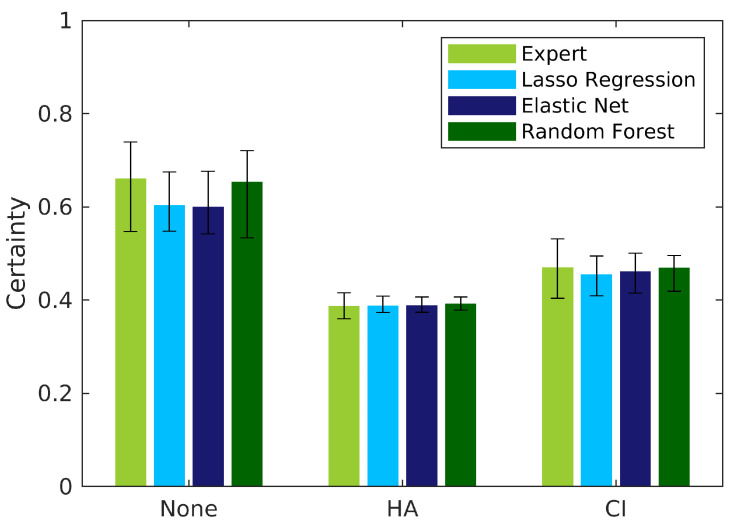
Median and interquartile ranges for certainty of classification in tree set III. Certainties of single CAFPAs are combined according to *rel-model* weights and propagated through the tree of comparison sets. Each bar represents patients that were classified to their respective category using expert-estimated or predicted CAFPAs (color-coded). Table A2 summarizes certainty results for the remaining tree sets and weights.

Finally, certainty for all categories and models in tree set III (*rel-model* weights) is depicted in Figure 10. Each bar displays the median and interquartile ranges of patients that were classified into the respective category based on CAFPAs predicted by the different models. For all categories, all (also individual) certainties are above the chance rate, which is 0.5 for the first and 0.25 for the second and third category, as HA and CI are classified from patients that were previously classified as needing a hearing device (two subsequent comparison sets). For the none category, the median for experts and random forest is slightly higher than for lasso regression and elastic net, but the corresponding interquartile range is also larger. Similar certainty relationships were found for all tree sets and weights (cf. Table A2). In all cases, the certainty values depend on the expert training distributions of two categories in each comparison set, as well as on the individual values relative to the classification threshold. That is, higher certainty for one model as compared to the others is due to the distribution of $p_{CAFPA}$ values as predicted by the model. In the current classification scheme, certainty could be improved if training distributions were narrower or with higher distance of the means of the distributions. With more (balanced) data in training, this could change; however, the training distributions can also be a property of the data set if the data set is already representative.

In summary, the classification in tree sets performs well, but needs to be improved towards use in clinical context. Depending on the tree set (and different underlying data properties), confusions between compared categories happen, but can be explained with the classification procedure and especially the data employed for training, which should include more severe hearing losses in the future. Only small differences occur between weights; therefore model-specific (*rel-model*) weights are most plausible to use because the knowledge about the importance of CAFPAs for classification is included and the generation approach should generalize to future estimation of model-specific weights when larger data sets are used. Regarding the choice of CAFPA prediction models, some differences were identified, but all work plausibly, and a decision should be kept until a larger, more balanced data set is included.

## 4. Discussion

The current study explored the feasibility of constructing a clinical decision support system (CDSS) for audiology based on Common Audiological Functional Parameters (CAFPAs) which is as interpretable as expert knowledge-based classification and as data-driven as machine learning-based classification. The feasibility of using predicted CAFPAs as compared to expert-estimated CAFPAs was investigated, which is an important prerequisite for the application of the CDSS to individual patients. The classification performance was evaluated in terms of comparable performance between expert-estimated and predicted CAFPAs, as well as in terms of the interpretability of the obtained classification.

### 4.1. Classification Based on Expert-Estimated vs. Model-Predicted CAFPAs

All three regression models for prediction of CAFPAs [33] performed generally similar to expert-estimated CAFPAs in the classification task, and can therefore be used in the CDSS. However, high and comparable performance between expert-estimated and predicted CAFPAs depends on the respective choice of weights defining the combination of CAFPAs. Hence, it is crucial to employ plausible weights in the classification. The criterion for investigating these weights (Y90 combinations) was chosen to represent a robust amount of CAFPA combinations, i.e., not only relying on one best performing combination, but also not on too many combinations. The resulting numbers ranged between a single CAFPA combination and nearly half of all possibilities (comparison set I for common weights of expert and random forest, cf. Figure 5), which is due to the definition of the criterion based on relative performance instead of a fixed number of best combinations. The former should provide better comparability between different prediction models and comparison sets.

The importance of CAFPAs for different comparison sets (as defined by Y90 criterion) is similar across the different prediction models, but different across comparison sets. This resulted in plausible CAFPAs in these diagnostic decisions, regarding the definition of CAFPAs but also the underlying measurements used in the model-building process for different CAFPAs in Saak et al. [33]. There, plausible relationships between CAFPAs and measurements were found by analysis of feature importance. In total, all CAFPAs contribute to high performance in at least one comparison set, which again confirms the choice of CAFPAs (as found in [31,32]). However, different subsets of CAFPAs also show similar performance, that is, it cannot be said that all CAFPAs provide additional information in all cases. However, by including more CAFPAs in the choice of weights for application to individual patients (cf. Section 3.2), the classification should be more robust towards changes in single CAFPAs. Therefore, the relative frequency of CAFPAs in common weight combinations between expert-estimated and predicted CAFPAs (*rel-model*) was chosen as the weights to be used in the classification, which also resulted in high classification performance.

### 4.2. Classification of Individual Patients

To classify individual patients with the CDSS, the comparison sets were combined to tree sets, and the weights from the first part were used to combine CAFPAs. In this setup, the classification was evaluated in terms of CAFPA patterns, differences between expert-estimated and predicted CAFPAs (accuracy), and certainty. While the overall performance was good, different classified categories based on expert-estimated or predicted CAFPAs occured in some cases. All results can be explained with the properties of the data, the classification method with its underlying training distributions, and the properties of the CAFPA prediction. According to Saak et al. [33], less extreme CAFPAs were predicted as compared to expert-estimated CAFPAs. In the classification task considered here, this effect was less pronounced due to the comparison of only two respective categories, but played a role for CAFPAs near the classification threshold [40]. Effectively, a shift of classification threshold (corresponding to using training distributions derived from the respective model-predicted CAFPAs) could compensate for that, but this would exploit knowledge that is not available in the real use case of a clinical decision support system. Instead, the accuracy should be increased if more training data for a larger and more balanced group of patients are available in the future [41]. This data could be employed in the derivation of CAFPA prediction models as well as in the estimation of the training distributions. Both aspects could also influence certainty of the CDSS’ decision: the former in terms of individual $p_{CAFPA}$ values being more correct relative to the classification threshold, and the latter when training distributions are more representative of real data, which could lead to narrower distributions and therefore better-separable categories and higher certainty. However, the current certainty could also be a property of the data if training distributions do not change with more training data.

In the use case of the CDSS applied to an individual new patient, the system would output its estimated category from every tree set, along with individual certainty. In addition, CAFPAs for the current patient would be displayed, which have been predicted based on the same input data from measurements that the expert has available for his or her own conclusion about the patient.

### 4.3. Interplay between Experts and CDSS and Interpretability

The interplay of experts and CDSS should provide optimal benefits towards precision medicine. Experts can draw their own conclusions and are, in addition, supported by the automatic decision and certainty provided by the CDSS, which can add objectivity backed up by data [1,2]. To trust the system, interpretability was stated as important, e.g., by [1,4]. In the current system, interpretability was considered in several aspects. First, the system was developed based on expert knowledge [5,6,7], the definition of CAFPAs was discussed with experts [30], and expert CAFPAs were estimated to provide a first link to audiological data [31] and to compare classification of CAFPAs to measurements [32]. The regression models of Saak et al. [33] were also established based on this expert knowledge. Second, the classification and prediction procedures allow insights into different steps and explanations regarding how results were generated [1,3,11,12], such as, for example, the analysis of expert-estimated vs. predicted CAFPAs relative to the classification threshold shown in the current paper, the analysis of CAFPAs important for classification (weights), or the analysis of measurements underlying the CAFPA predictions (feature importance) by [33]. Third, the final tool provides interpretability when applied to individual patients, for example by visualization [1].

In this use case, in addition to the general output, such as classified category and certainty, different interpretable components could be presented to the expert user. The CAFPAs are not only used as an intermediate layer for classification, but are also provided as a visualization to give an abstract overview of the current patient’s auditory system. By looking at the measurement results and comparing it to the shown CAFPAs, the expert can estimate on his/her own if the CAFPAs are plausible. This could be further supported by presenting importance (by means of weights) of different CAFPAs along with their relationship to the measurements, for example also presenting only those measurements that contributed most to the current classification.

### 4.4. CDSS for Audiology Based on CAFPAs

In summary, the presented CDSS based on predicted CAFPAs was shown to be feasible in terms of functionality for individual patients, and it provides good classification performance as well as interpretability. However, potential for improvement lies in the integration of additional clinical-audiological databases, on the one hand to generally increase the number of included patients towards "Big Data", and on the other hand to better represent severe patient cases in the CDSS. It is expected that the approach generalizes to other data sets, and if the number of patients increases in the future, it could be investigated if more sophisticated machine learning methods improve classification performance. For example, Mousavi et al. [42] developed a classification approach that can deal with CAFPAs as continuous input variable, as well as with multiple findings being true for a patient. On the basis of the current CDSS framework, the integration of additional databases can be evaluated.

### 4.5. Towards Integration of Clinical Databases

To enable integration of additional clinical databases into the CDSS based on CAFPAs, every new database needs to be linked to CAFPAs; hence, CAFPAs need to be estimated for patients based on the respective clinical test battery of audiological measurements. The current prediction by Saak et al. [33] only includes measurements from the database of Hörzentrum Oldenburg as described in [34]. This prediction can be updated to cover only those measurements that are common between the current and a to-be-included database, and then be applied to predict CAFPAs based on this information. For additional measurements, such as, for example, the Freiburg monosyllabic speech test [19], which is commonly used for indication of hearing devices according to German clinical guidelines [18], or the matrix sentence test [20,21,22,23], additional expert knowledge could be collected to link these measurements to CAFPAs. Compared to the expert survey of [31], only a limited number of distinct patient profiles could be shown to the experts to increase efficiency, and the experts could be asked to update the predicted CAFPAs based on fewer measurements instead of estimating CAFPAs from scratch. As an additional consistency check independent from the CAFPA concept, consistency between databases could be investigated by the use of models, for example in the context of speech intelligibility, where different tests are used for different purposes or in different countries. If data standards for audiology get established in the future, for example, in the context of the HiGHmed initiative [13] and open electronic health records (openEHR), the integration of additional databases would be facilitated. However, such a process takes time and different measurements could still be performed in different clinics, which makes a combination of the CAFPA concept with data standardization approaches most promising for obtaining a largest-possible data basis for a clinical decision support system for audiology.

## 5. Conclusions

The main conclusion of this work is that it is feasible to obtain an interpretable yet data-driven clinical decision support system for audiology. This was achieved by combining previous approaches of audiological classification based on expert-estimated CAFPAs and regression models for prediction of CAFPAs, which were built based on expert knowledge. Including the data-driven prediction of CAFPAs in the CDSS allows classification of new, individual patients, which represents the typical use case of a CDSS, and was not possible before combining classification and data-driven prediction of CAFPAs.

Predicted CAFPAs are valid to be used in the CDSS, and classification performance is high except for some differences between classification based on expert-estimated vs. predicted CAFPAs, which can be explained by properties of prediction and the employed database. The CDSS is interpretable in terms of providing insights into the classification process as well as during application by experts, especially by the use of CAFPAs as an interpretable intermediate layer.

The current system will be used in the future as an evaluation framework for the integration of additional clinical databases. In the long run, the classification procedures itself could be further investigated and potentially improved.

## Figures and Tables

**Table 1:** Accuracy for different tree sets, weights, and CAFPA prediction models. Numbers in parentheses indicate that the choice of weights was not based on Y90 combinations but on one single CAFPA combination (cf. Figure 5).

Weights	Model	Tree Set I	Tree Set II	Tree Set III
*uniform*	Lasso regression	0.67	0.58	0.70
*uniform*	Elastic net	0.67	0.57	0.69
*uniform*	Random forest	0.66	0.60	0.71
*rel-model*	Lasso regression	(0.64)	(0.59)	0.75
*rel-model*	Elastic net	(0.64)	0.61	0.75
*rel-model*	Random forest	(0.67)	(0.62)	0.78
*rel-all*	Lasso regression	(0.74)	(0.59)	0.78
*rel-all*	Elastic net	(0.73)	(0.58)	0.77
*rel-all*	Random forest	(0.71)	(0.58)	0.78

## Data Availability

The data and code presented in this study are openly available under https://doi.org/10.5281/zenodo.5938631 (accessed on 27 December 2021). The predicted CAFPAs were generated according to the published code of [33].

## Supplementary Materials

The following are available online at https://www.mdpi.com/article/10.3390/diagnostics12020463/s1, Figure S1: CAFPA patterns (median and interquartile ranges) of patients classified in all tree sets for expert and all models (rows) using different weights. Tree set and weights are indicated at the top left corner of each page. *N* indicates the number of included patients.

## Abbreviations

The following abbreviations are used in this manuscript:

ACALOS	Adaptive categorical loudness scaling
Acc	Accuracy
BTE	Behind-the-ear hearing aid
*c*	Classified category (index)
CAFPAs	Common Audiological Functional Parameters
$C_{A1}$-$C_{A4}$	Hearing threshold-related CAFPAs
$C_{U1}$-$C_{U2}$	Supra-threshold CAFPAs
$C_{B}$	Binaural CAFPA
$C_{N}$	Neural CAFPA
$C_{C}$	Cognitive CAFPA
$C_{E}$	Socio-economic CAFPA
CDSS	Clinical decision support system
Cert	Certainty
CI	Cochlear implant
cond	Conductive hearing loss
CS	Comparison set
Device	Any hearing device
GÖSA	Goettingen sentence test
HA	Hearing aid
HI	Hearing impaired
high	High-frequency hearing loss
HiGHmed	Heidelberg–Göttingen–Hannover Medical Informatics
ITE	In-the-ear hearing aid
*N*	Number of patients
NH	Normal hearing
None	No hearing device
openEHR	Open electronic health record
*p*	Probability
$p_{CAFPA}$	CAFPA value [0 1]
recr	Recruitment
*rel-all*	Weights common for all models
*rel-model*	Weights derived for different prediction models
Sens	Sensitivity
Spec	Specificity
SWI	Scheuch–Winkler index
*uniform*	Uniform weights
*Y*	Youden index
Y90	Youden index criterion, values higher than 90 % of max(Y)

## Appendix A

### Appendix A.1. Confusion Matrices for All Weights

**Table A1:** Confusion matrices (absolute numbers) for all tree sets and weights. For each weight, the data are organized as in Figure 8. Category numbers correspond to the respective categories of each tree set.

			Tree Set I			Tree Set II			Tree Set III		
Weights	Model	Category	Category (Expert)								
		(Predicted)	1	2	3	1	2	3	1	2	3
*uniform*	Lasso regression	3	1	20	37	16	61	98	0	7	17
		2	31	99	21	16	14	4	29	79	23
		1	26	5	0	26	4	1	71	14	0
	Elastic net	3	2	20	37	17	62	98	0	7	17
		2	30	99	21	15	13	4	29	77	23
		1	26	5	0	26	4	1	71	16	0
	Random forest	3	1	30	37	17	64	100	0	9	22
		2	21	86	21	5	8	2	28	77	18
		1	36	8	0	36	7	1	72	14	0
*rel-model*	Lasso regression	3	2	8	24	13	55	100	0	4	17
		2	26	102	44	15	14	9	21	100	22
		1	28	6	0	28	5	1	62	14	0
	Elastic net	3	1	5	21	11	53	102	0	4	18
		2	27	105	47	17	16	7	20	99	21
		1	28	6	0	28	5	1	63	15	0
	Random forest	3	2	23	39	15	53	100	0	6	25
		2	19	86	29	6	14	10	18	96	14
		1	35	7	0	35	7	0	65	16	0
*rel-all*	Lasso regression	3	2	17	37	19	60	96	0	4	18
		2	24	112	13	7	17	6	20	101	19
		1	29	6	0	29	2	4	68	10	0
	Elastic net	3	2	17	35	21	62	97	0	5	18
		2	26	112	15	7	15	5	20	100	19
		1	27	6	0	27	2	4	68	10	0
	Random forest	3	2	26	36	15	61	92	0	8	23
		2	19	101	14	6	14	10	18	95	14
		1	34	8	0	34	4	4	70	12	0

### Appendix A.2. Certainty for All Tree Sets and Weights

**Table A2:** Certainty (median and interquartile range) for all tree sets and weights. For each weight, the data are organized as in Figure 10. Category numbers correspond to the respective categories of each tree set.

			Classified Category		
Tree Set	Weights	Model	1	2	3
			Median [Interquartile Range]		
I	*uniform*	Expert	0.66 [0.57 0.71]	0.33 [0.30 0.36]	0.44 [0.40 0.48]
		Lasso regression	0.60 [0.55 0.62]	0.34 [0.32 0.37]	0.42 [0.39 0.44]
		Elastic net	0.59 [0.55 0.62]	0.34 [0.32 0.37]	0.42 [0.39 0.45]
		Random forest	0.64 [0.59 0.66]	0.34 [0.31 0.36]	0.42 [0.40 0.47]
	*rel-model*	Expert	0.67 [0.56 0.73]	0.33 [0.31 0.37]	0.42 [0.38 0.46]
		Lasso regression	0.60 [0.55 0.65]	0.36 [0.32 0.40]	0.39 [0.38 0.42]
		Elastic net	0.60 [0.54 0.64]	0.36 [0.32 0.39]	0.41 [0.38 0.43]
		Random forest	0.67 [0.59 0.69]	0.43 [0.41 0.46]	0.53 [0.44 0.63]
	*rel-all*	Expert	0.66 [0.57 0.72]	0.42 [0.38 0.47]	0.65 [0.48 0.74]
		Lasso regression	0.59 [0.54 0.65]	0.42 [0.39 0.45]	0.47 [0.42 0.60]
		Elastic net	0.60 [0.55 0.65]	0.42 [0.39 0.45]	0.47 [0.43 0.62]
		Random forest	0.65 [0.59 0.68]	0.43 [0.40 0.47]	0.52 [0.42 0.62]
II	*uniform*	Expert	0.66 [0.57 0.71]	0.31 [0.27 0.34]	0.40 [0.33 0.43]
		Lasso regression	0.60 [0.55 0.62]	0.27 [0.26 0.29]	0.40 [0.33 0.43]
		Elastic net	0.59 [0.55 0.62]	0.27 [0.26 0.29]	0.40 [0.33 0.43]
		Random forest	0.64 [0.59 0.66]	0.27 [0.27 0.30]	0.41 [0.34 0.44]
	*rel-model*	Expert	0.67 [0.56 0.73]	0.32 [0.30 0.38]	0.42 [0.34 0.45]
		Lasso regression	0.60 [0.55 0.65]	0.30 [0.29 0.31]	0.43 [0.37 0.47]
		Elastic net	0.60 [0.54 0.64]	0.32 [0.30 0.32]	0.45 [0.38 0.50]
		Random forest	0.67 [0.59 0.69]	0.29 [0.29 0.31]	0.43 [0.36 0.46]
	*rel-all*	Expert	0.66 [0.57 0.72]	0.35 [0.32 0.43]	0.40 [0.34 0.46]
		Lasso regression	0.59 [0.54 0.65]	0.29 [0.28 0.42]	0.41 [0.35 0.45]
		Elastic net	0.60 [0.55 0.65]	0.29 [0.28 0.40]	0.41 [0.35 0.45]
		Random forest	0.65 [0.59 0.68]	0.29 [0.28 0.31]	0.42 [0.36 0.45]
III	*uniform*	Expert	0.59 [0.54 0.69]	0.36 [0.34 0.37]	0.46 [0.41 0.52]
		Lasso regression	0.58 [0.54 0.64]	0.37 [0.36 0.38]	0.43 [0.40 0.48]
		Elastic net	0.57 [0.53 0.64]	0.37 [0.36 0.38]	0.43 [0.41 0.47]
		Random forest	0.60 [0.53 0.67]	0.37 [0.36 0.38]	0.45 [0.39 0.46]
	*rel-model*	Expert	0.66 [0.55 0.74]	0.39 [0.36 0.42]	0.47 [0.40 0.53]
		Lasso regression	0.60 [0.55 0.68]	0.39 [0.37 0.41]	0.46 [0.41 0.49]
		Elastic net	0.60 [0.54 0.68]	0.39 [0.37 0.41]	0.46 [0.42 0.50]
		Random forest	0.65 [0.53 0.72]	0.39 [0.38 0.41]	0.47 [0.42 0.50]
	*rel-all*	Expert	0.63 [0.54 0.72]	0.38 [0.36 0.39]	0.49 [0.43 0.53]
		Lasso regression	0.60 [0.55 0.67]	0.38 [0.37 0.40]	0.46 [0.42 0.50]
		Elastic net	0.60 [0.54 0.68]	0.38 [0.37 0.40]	0.47 [0.42 0.50]
		Random forest	0.64 [0.53 0.71]	0.39 [0.37 0.40]	0.47 [0.42 0.50]
