# Supplementary material for: Interpretable Clinical Decision Support System for Audiology Based on Predicted Common Audiological Functional Parameters (CAFPAs)
Source: Diagnostics (Basel). 2022 Feb 11;12(2):463. doi: 10.3390/diagnostics12020463 (PMC8870744; doi:10.3390/diagnostics12020463)
Supplement: Supplementary file 1 [file diagnostics-12-00463-s001.zip › Figure S1.pdf]

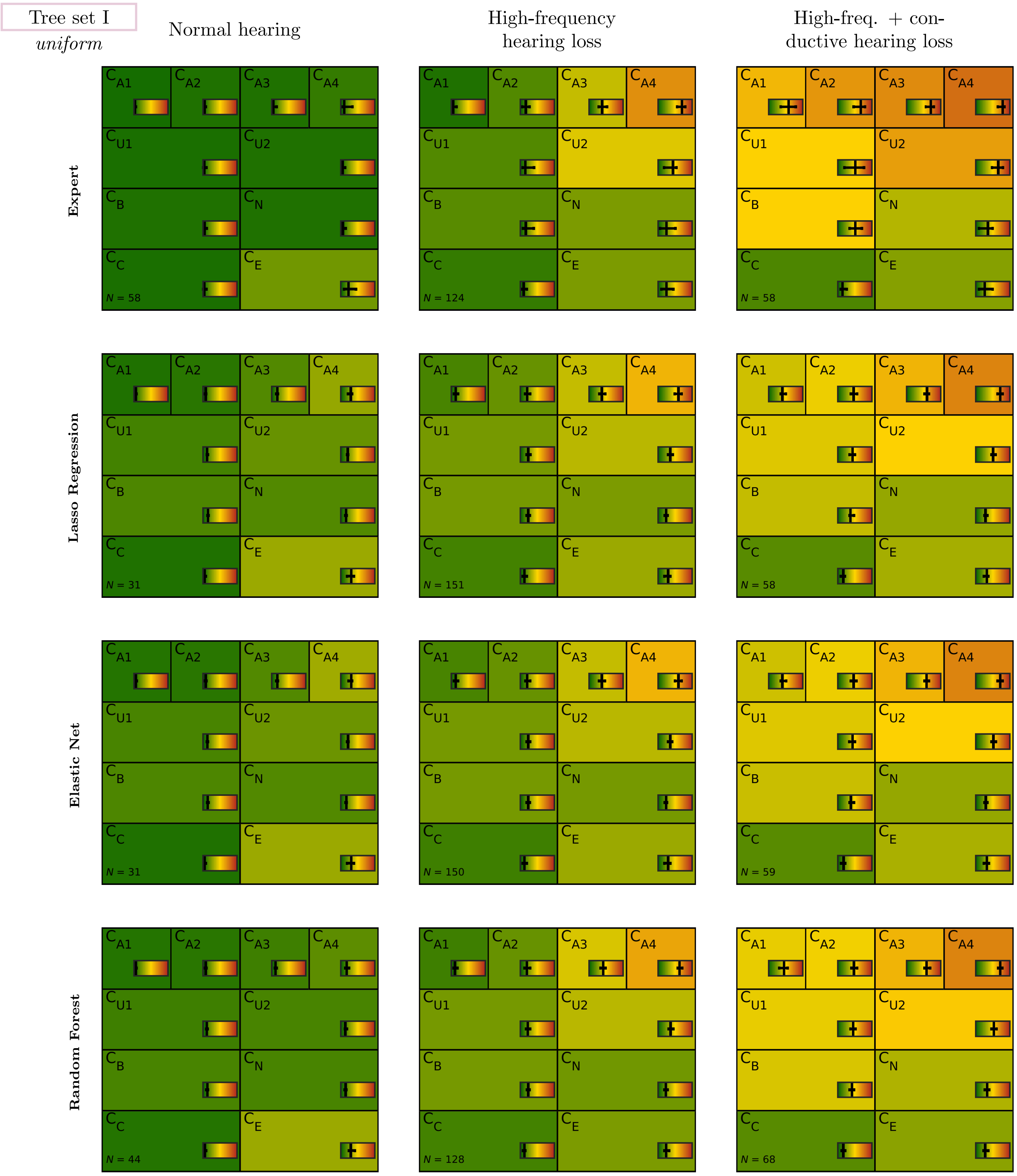

Tree set I
uniform
Normal hearing
High-frequency hearing loss
High-freq. + conductive hearing loss
Expert
Lasso Regression
Elastic Net
Random Forest
CA1
CA2
CA3
CA4
CU1
CU2
CB
CN
CC
CE
N = 58
N = 124
N = 58
N = 31
N = 151
N = 58
N = 31
N = 150
N = 59
N = 44
N = 128
N = 68

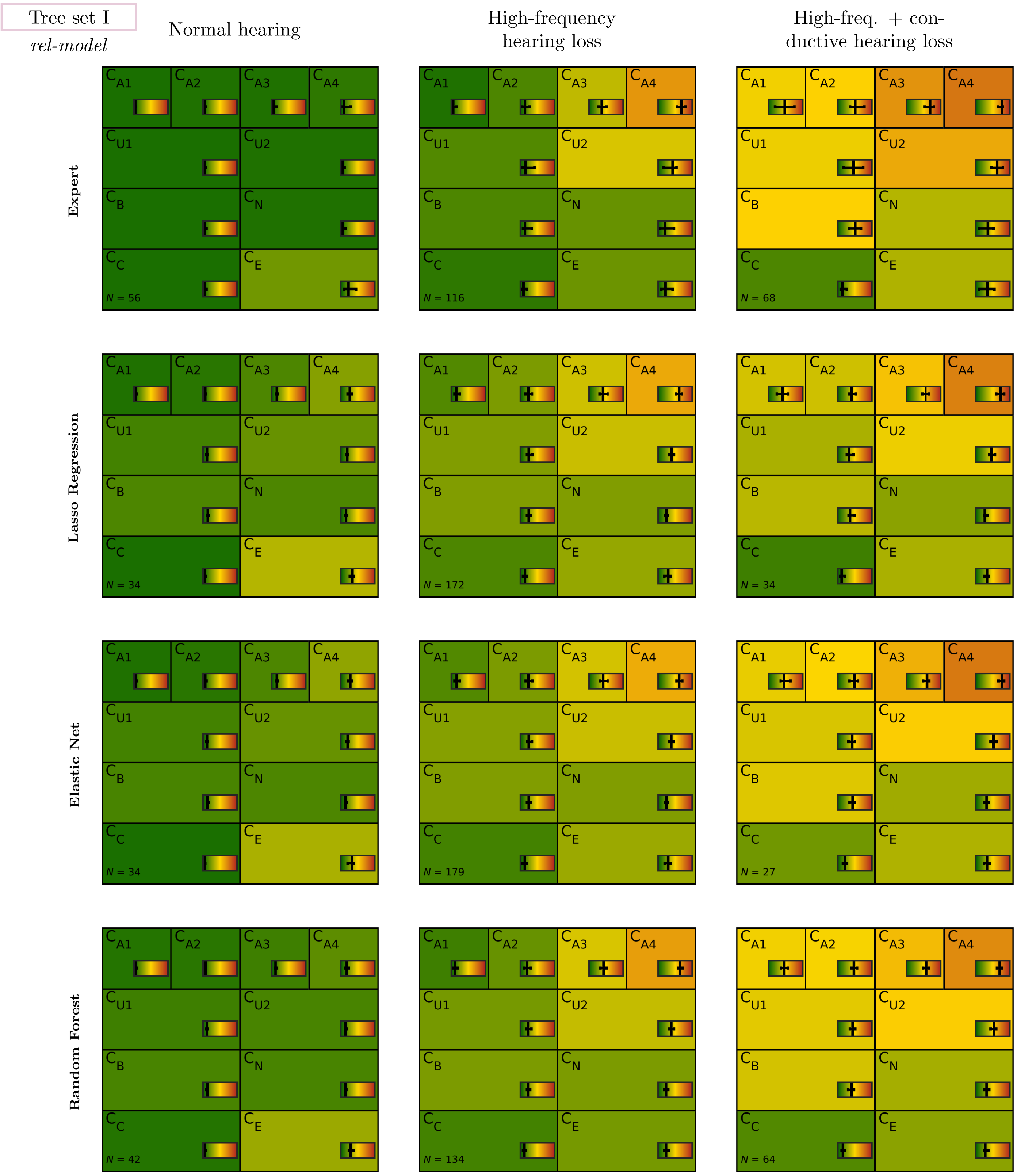
Tree set I
rel-model
Normal hearing
High-frequency hearing loss
High-freq. + conductive hearing loss
Expert
Lasso Regression
Elastic Net
Random Forest
C_{A1}
C_{A2}
C_{A3}
C_{A4}
C_{U1}
C_{U2}
C_B
C_N
C_C
C_E
N = 56
N = 116
N = 68
N = 34
N = 172
N = 34
N = 34
N = 179
N = 27
N = 42
N = 134
N = 64

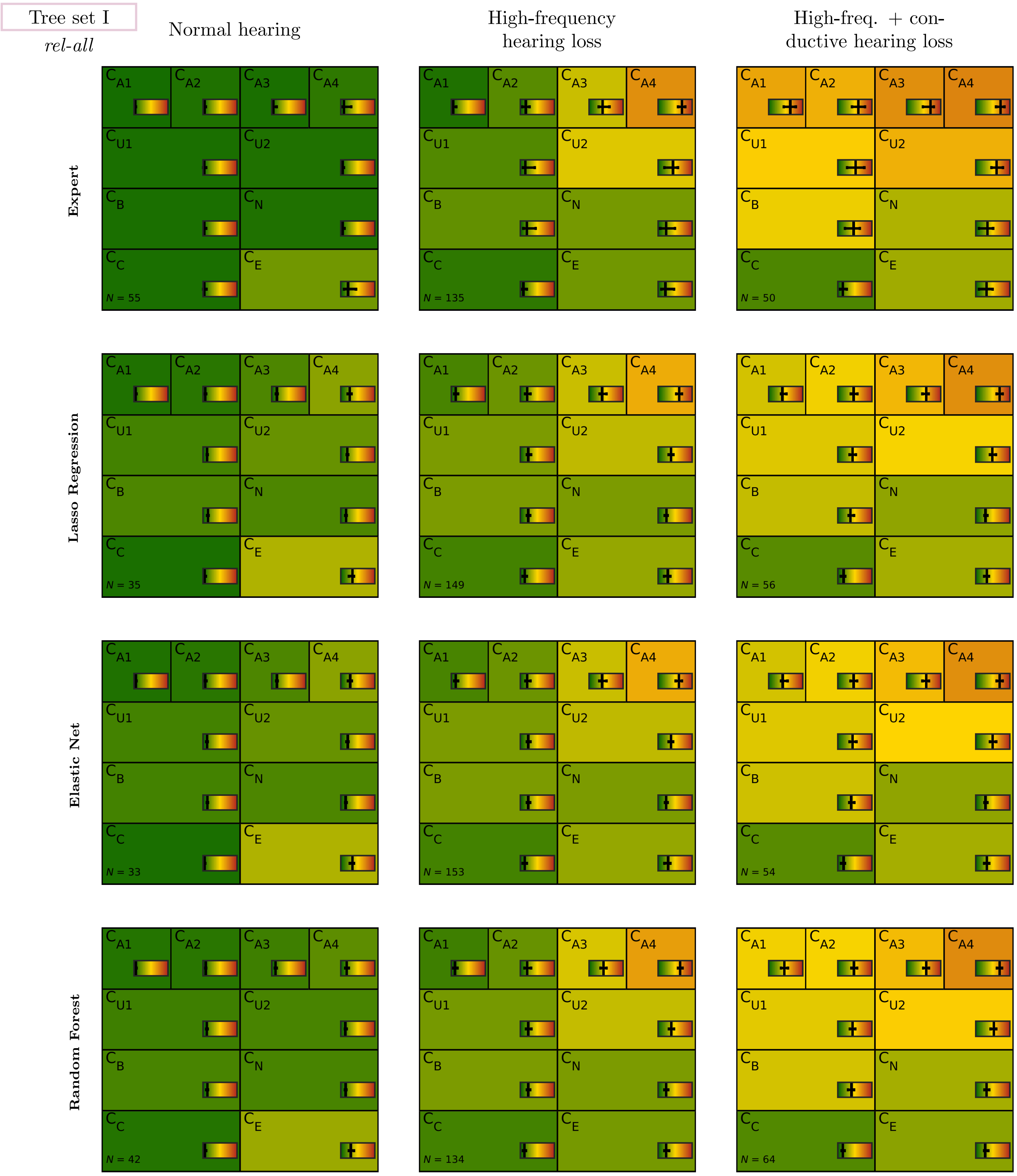
Tree set I
rel-all
Normal hearing
High-frequency hearing loss
High-freq. + conductive hearing loss
Expert
Lasso Regression
Elastic Net
Random Forest
C_A1 C_A2 C_A3 C_A4
C_U1 C_U2
C_B C_N
C_C C_E
N = 55
N = 135
N = 50
N = 35
N = 149
N = 56
N = 33
N = 153
N = 54
N = 42
N = 134
N = 64

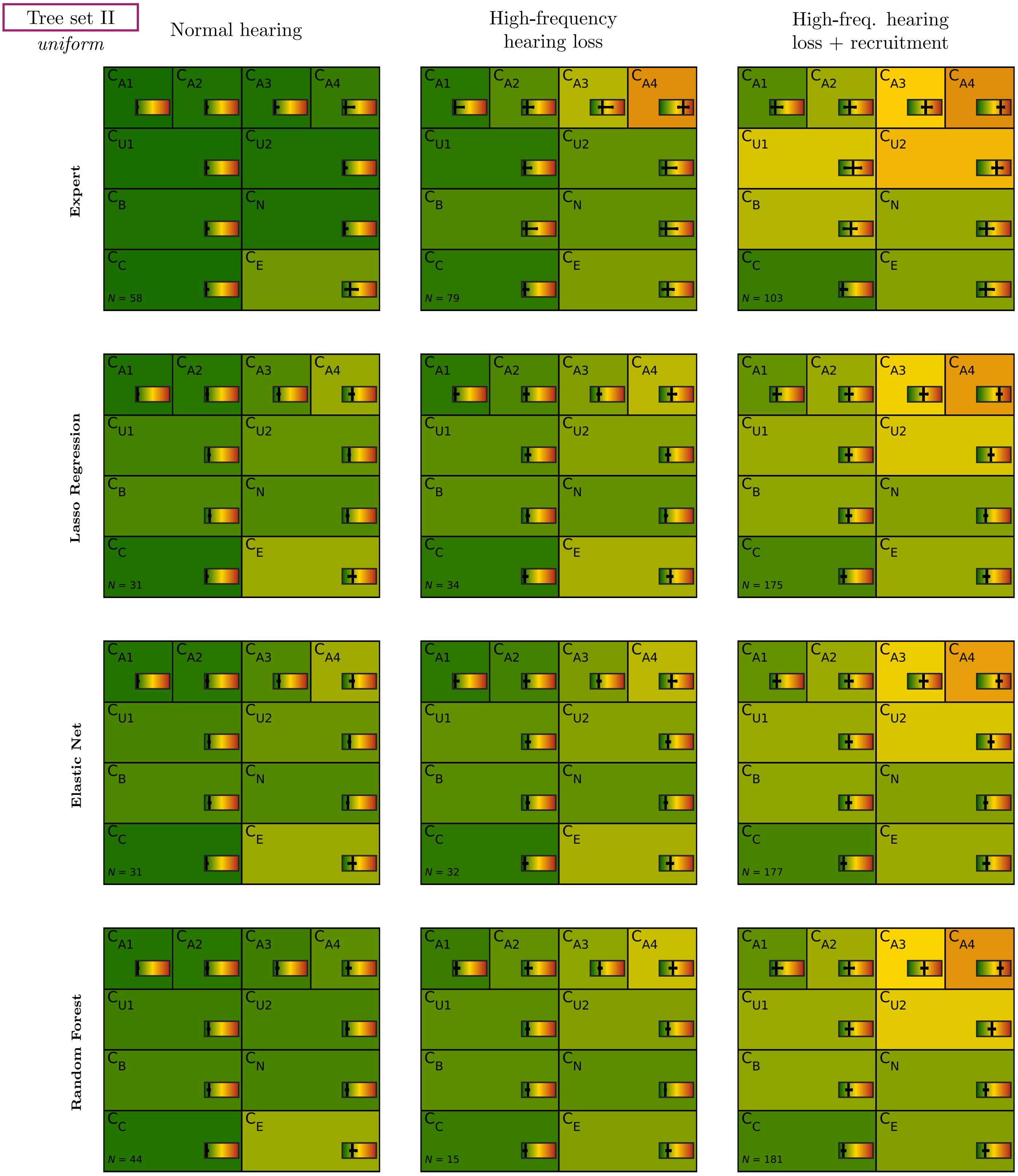

Tree set II
uniform
Normal hearing
High-frequency hearing loss
High-freq. hearing loss + recruitment
Expert
Lasso Regression
Elastic Net
Random Forest
C_A1 C_A2 C_A3 C_A4
C_U1 C_U2
C_B C_N
C_C C_E
N = 58
N = 79
N = 103
N = 31
N = 34
N = 175
N = 31
N = 32
N = 177
N = 44
N = 15
N = 181

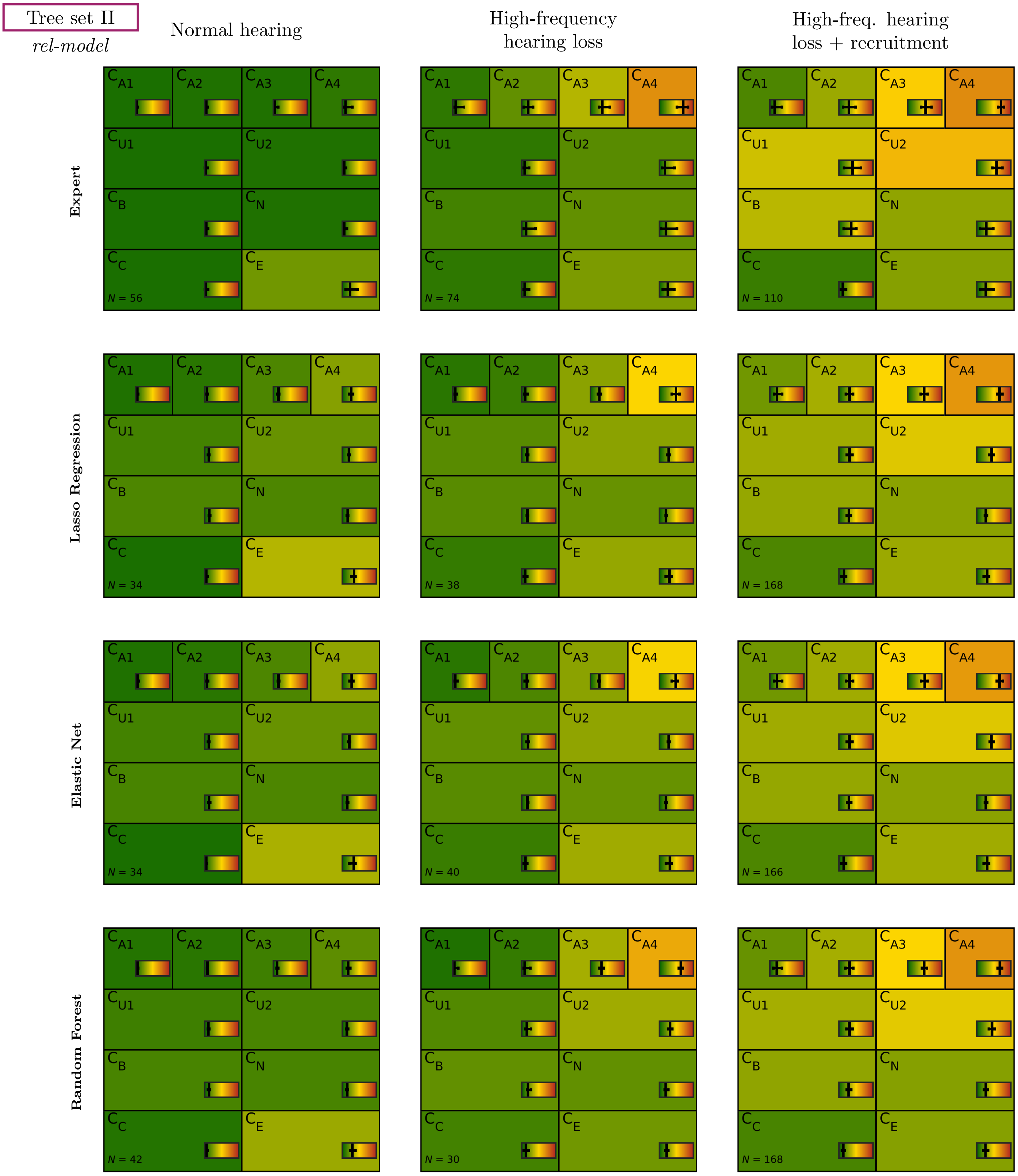
Tree set II
rel-model
Normal hearing
High-frequency hearing loss
High-freq. hearing loss + recruitment
Expert
Lasso Regression
Elastic Net
Random Forest
C_A1 C_A2 C_A3 C_A4
C_U1 C_U2
C_B C_N
C_C C_E
N = 56
N = 74
N = 110
N = 34
N = 38
N = 168
N = 34
N = 40
N = 166
N = 42
N = 30
N = 168

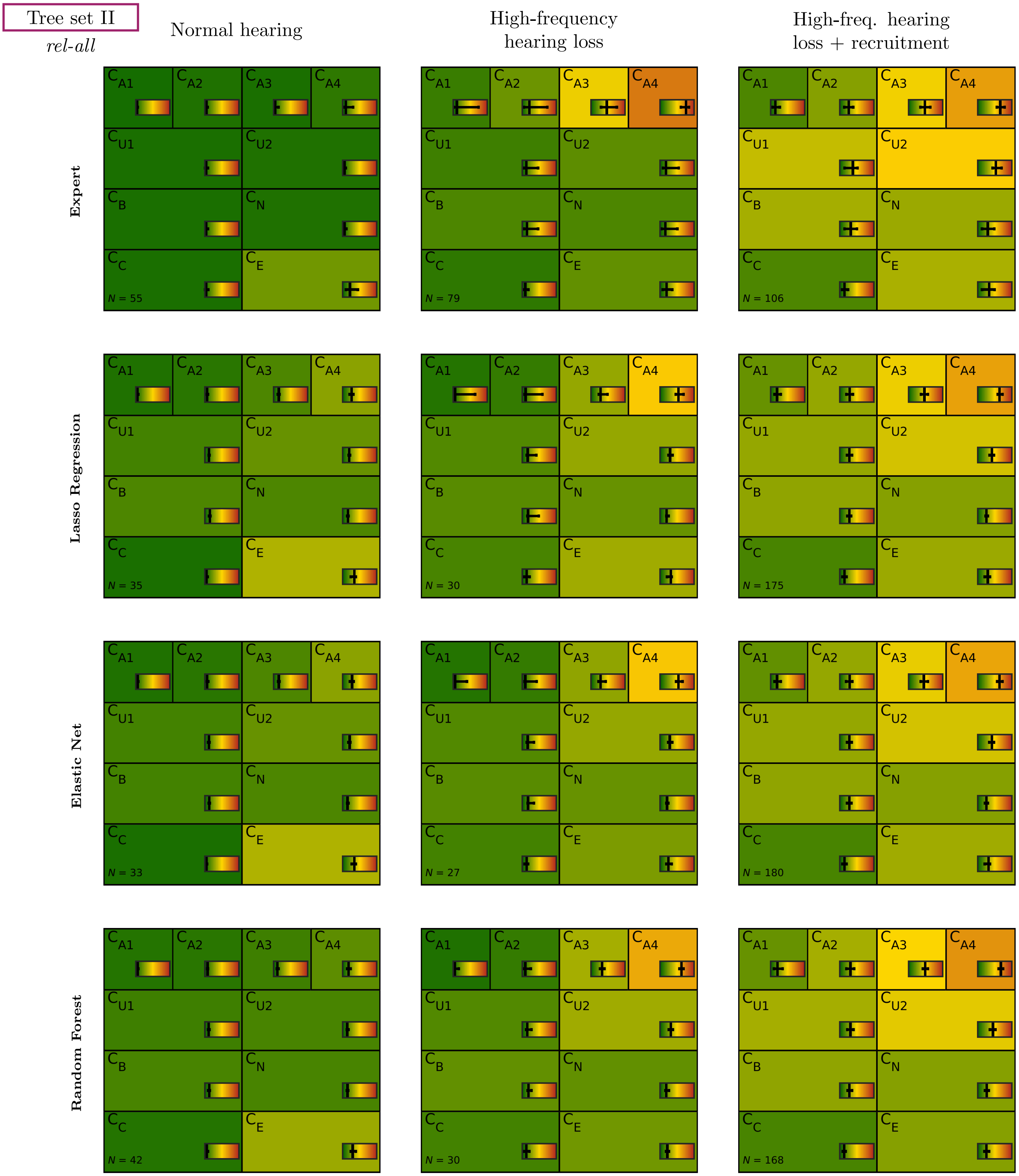
Tree set II
rel-all
Normal hearing
High-frequency hearing loss
High-freq. hearing loss + recruitment
Expert
Lasso Regression
Elastic Net
Random Forest
C_A1 C_A2 C_A3 C_A4
C_U1 C_U2
C_B C_N
C_C C_E
N = 55
N = 79
N = 106
N = 35
N = 30
N = 175
N = 33
N = 27
N = 180
N = 42
N = 30
N = 168

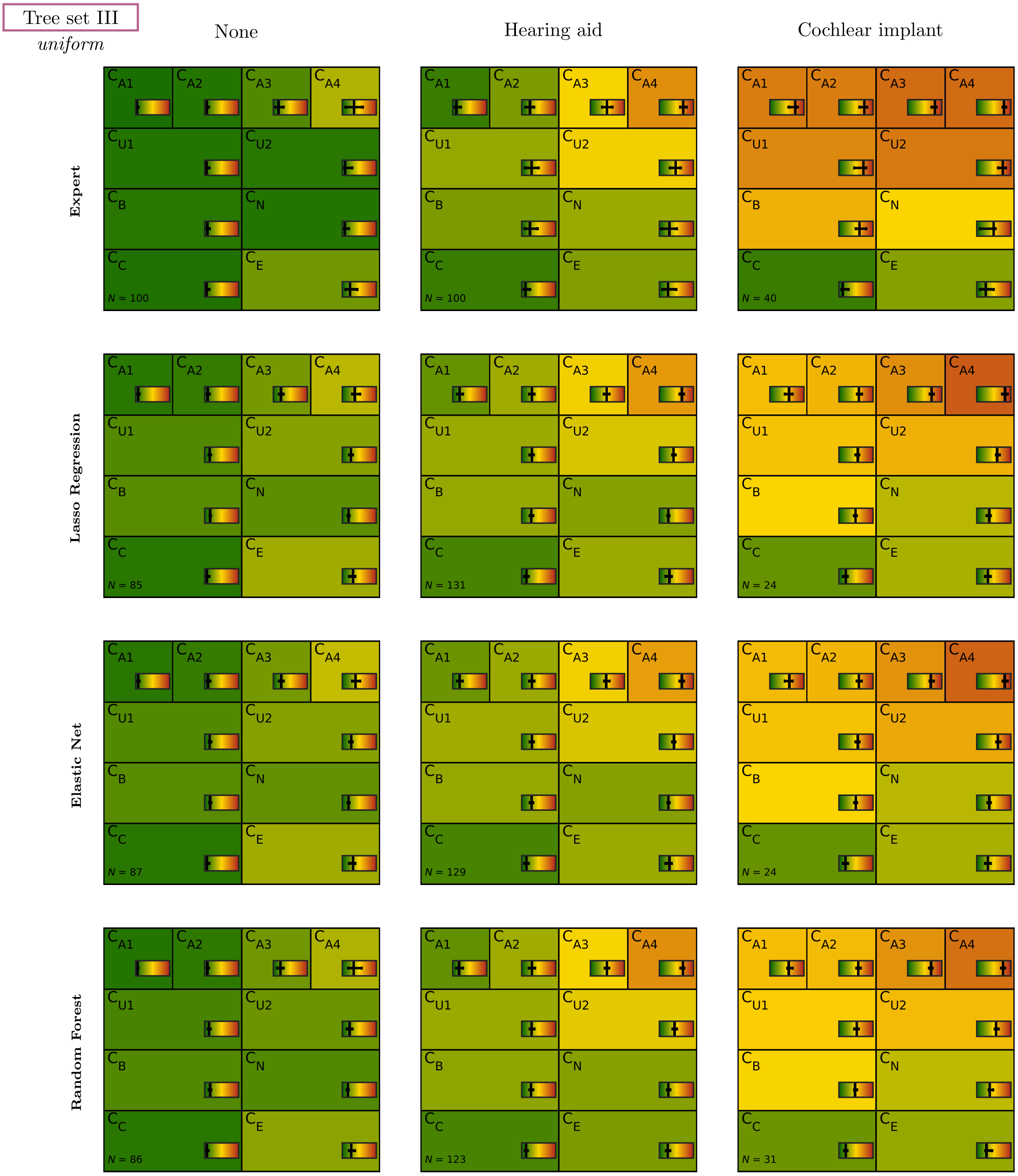
Tree set III
uniform
None
Hearing aid
Cochlear implant
Expert
Lasso Regression
Elastic Net
Random Forest
C A1
C A2
C A3
C A4
C U1
C U2
C B
C N
C C
C E
N = 100
N = 100
N = 40
N = 85
N = 131
N = 24
N = 87
N = 129
N = 24
N = 86
N = 123
N = 31

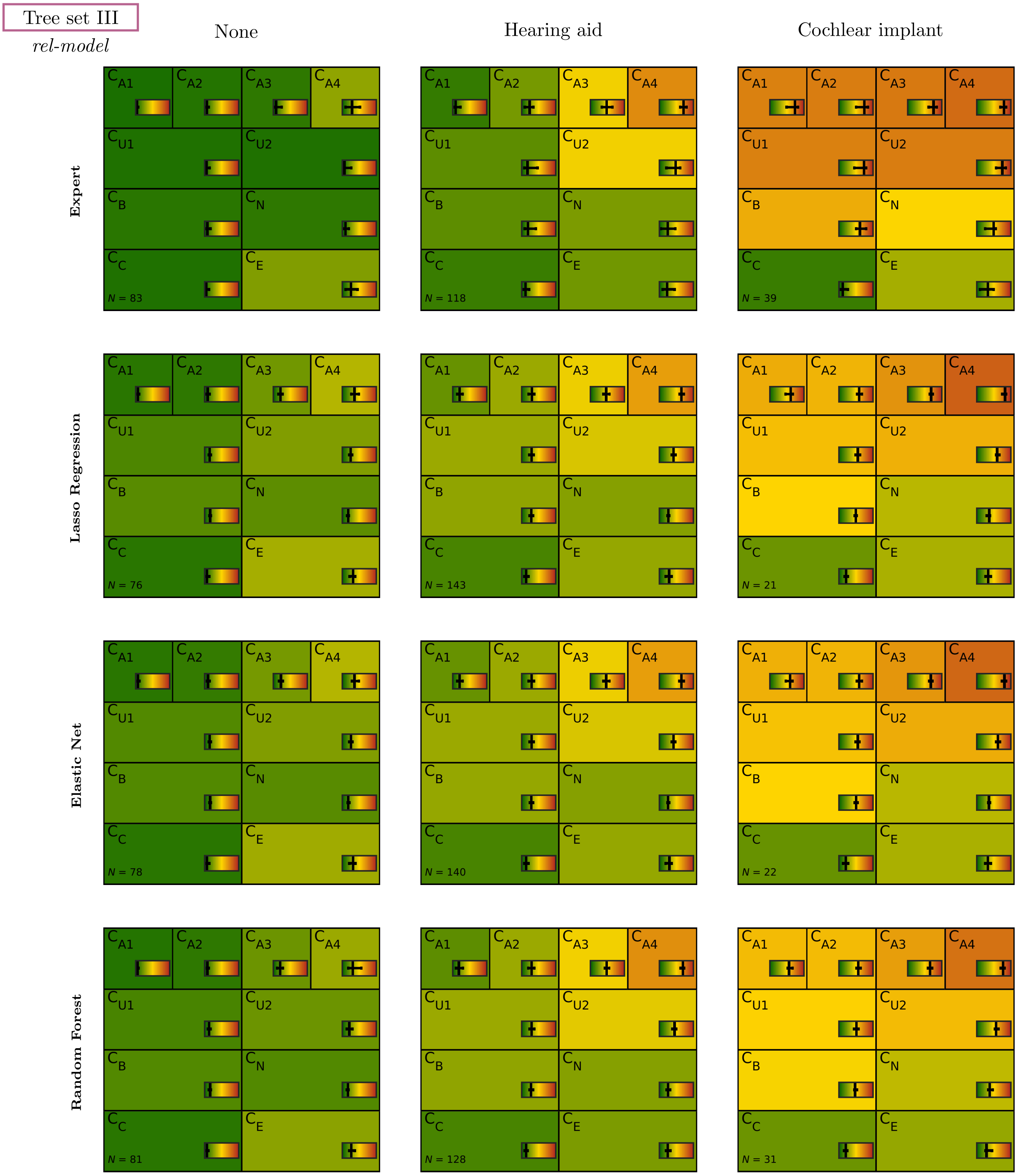
Tree set III
rel-model
None
Hearing aid
Cochlear implant
Expert
Lasso Regression
Elastic Net
Random Forest
CA1
CA2
CA3
CA4
CU1
CU2
CB
CN
CC
CE
N = 83
N = 118
N = 39
N = 76
N = 143
N = 21
N = 78
N = 140
N = 22
N = 81
N = 128
N = 31

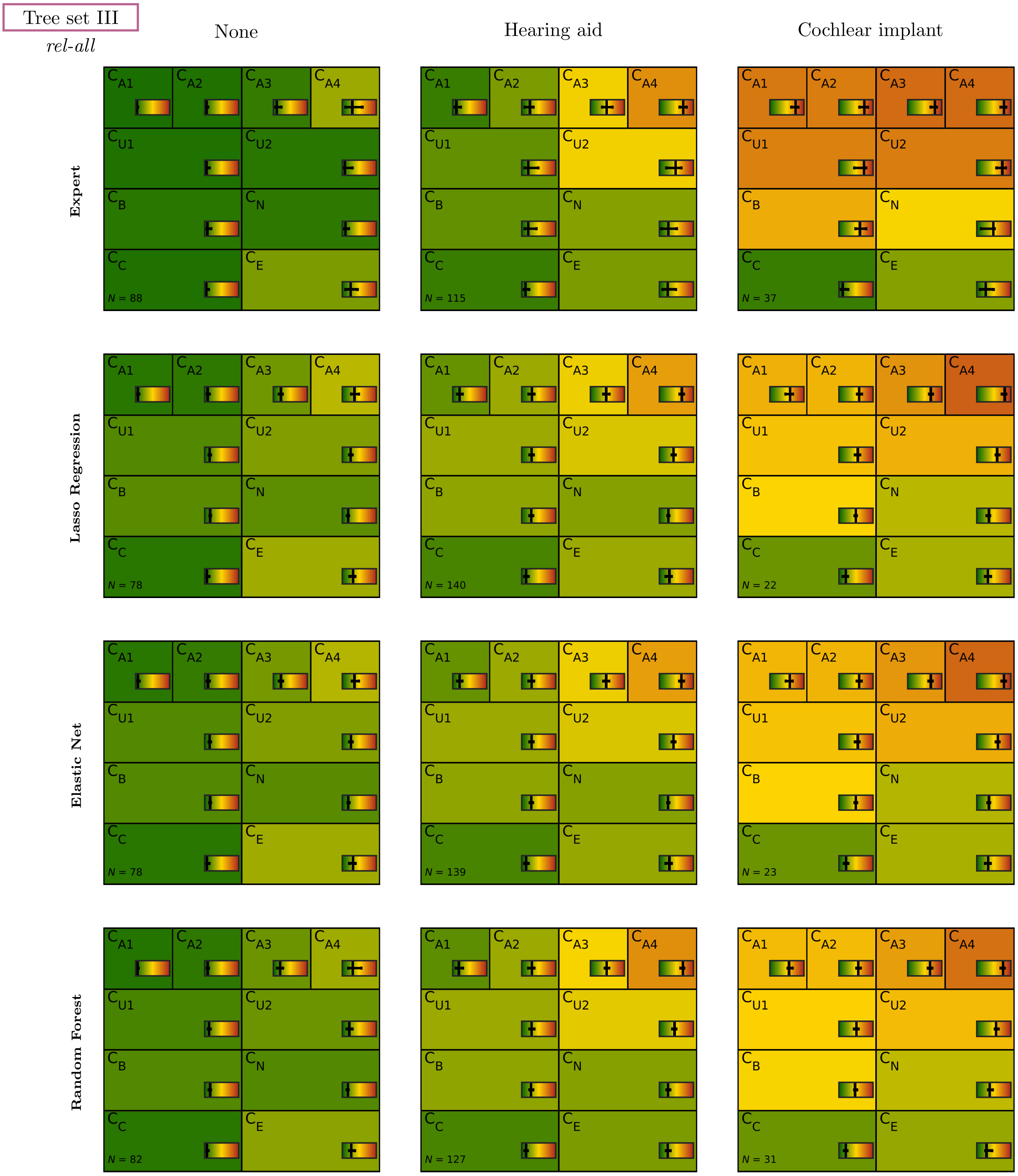

Figure S1. CAFPA patterns (median and interquartile ranges) of patients classified in all tree sets for expert and all models (rows) using different weights. Tree set and weights are indicated at the top left corner of each page. *N* indicates the number of included patients.
